# Tuning the stereo-hindrance of a curcumin scaffold for the selective imaging of the soluble forms of amyloid beta species†
†Electronic supplementary information (ESI) available. See DOI: 10.1039/c7sc02050c


**DOI:** 10.1039/c7sc02050c

**Published:** 2017-09-15

**Authors:** Yuyan Li, Jian Yang, Hongwu Liu, Jing Yang, Lei Du, Haiwei Feng, Yanli Tian, Jianqin Cao, Chongzhao Ran

**Affiliations:** a Molecular Imaging Laboratory , MGH/MIT/HMS Athinoula A. Martinos Center for Biomedical Imaging , Department of Radiology , Massachusetts General Hospital/Harvard Medical School , Charlestown , Massachusetts 02129 , USA; b School of Pharmacy , China Pharmaceutical University , Nanjing , 210009 , China; c College of Pharmaceutical Sciences , Soochow University , Suzhou , 215006 , China; d Department of Parasitology , Zhongshan School of Medicine , Sun Yat-Sen University , Guangzhou , P. R. China

## Abstract

Amyloid peptides and proteins are associated with the pathologies of numerous diseases. In the progression of a disease, amyloids exist in soluble and insoluble forms, which are the dominant species at different stages of the disease and they have different degrees of toxicity. However, differentiating between the soluble and insoluble forms is very challenging with small molecule probes due to multiple obstacles that need to be overcome. Inspired by the recognition principle of antibodies for sAβ, we hypothesized that the accessibility/tightness of soluble and insoluble amyloids could be utilized to design imaging probes to recognize different amyloid forms and the stereo-hindrance tuning strategy could be used to design imaging probes for selectively detecting the soluble amyloid beta (sAβ) species in Alzheimer’s disease (AD). Herein, we demonstrated that tuning the stereo-hindrance of the phenoxy-alkyl chains at the 4-position of a curcumin scaffold could lead to certain selectivity for sAβ over insoluble Aβs (insAβ). Among the designed compounds, **CRANAD-102** showed a 68-fold higher affinity for sAβ than for insAβ (7.5 ± 10 nM *vs.* 505.9 ± 275.9 nM). Moreover, our imaging data indicated that **CRANAD-102** was indeed capable of detecting sAβ *in vivo* using 4 month old APP/PS1 mice, in which sAβ is the predominant species in the brain. In addition, we also demonstrated that **CRANAD-102** could be used to monitor the increase in sAβ loading from the ages of 4 months old to 12 months old. We believe that **CRANAD-102** can be a useful probe for selectively detecting sAβ species in AD and that our probe designing strategy can be applied to other amyloids and will have tremendous impact on AD drug development and other amyloid research.

## Abstract 2

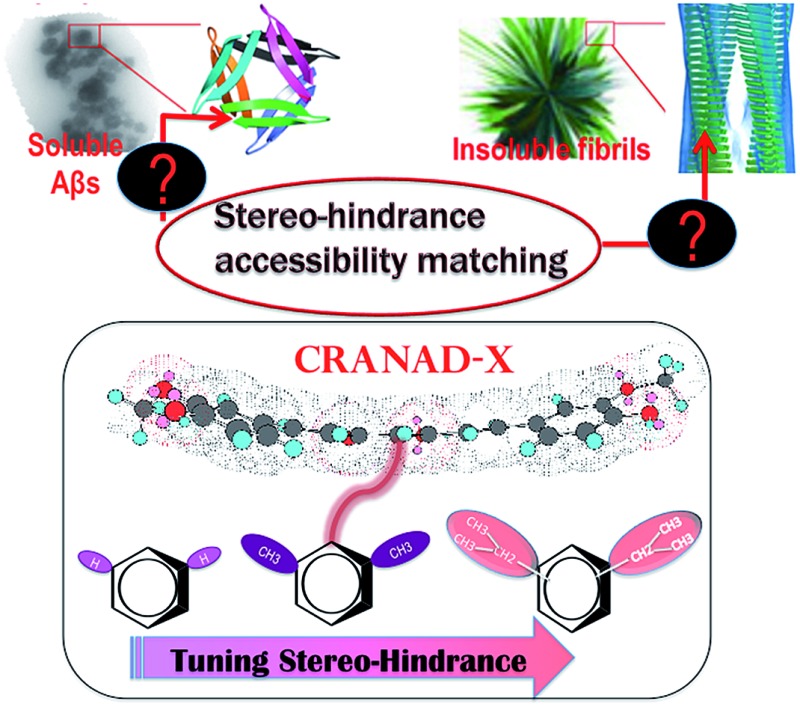
We demonstrate that tuning the stereo-hindrance of the phenoxy-alkyl chains at the 4-position of a curcumin scaffold could lead to certain selectivity for soluble Aβs over insoluble Aβs.

## Introduction

Amyloids are a large family of proteins/peptides that are prone to aggregate into large assemblies, which are usually harmful to human health. The most studied amyloids include amyloid beta (Aβ) and tau in Alzheimer’s disease (AD), α-synuclein in Parkinson’s disease, amylin in type II diabetes, and TDP-43 in amyotrophic lateral sclerosis.^1^–^4^ Amyloid is characterized by a cross-beta sheet quaternary structure,^5^–^7^ and it exists in soluble and insoluble forms in solutions and in tissues.^8^–^10^ Recent evidence suggests that the soluble and insoluble forms of amyloids have different toxicities,^8^–^11^ and they are the dominant species at different stages in the progression of the disease. In particular, the soluble form is likely to be the predominant species in the early stage of the disease,^8^–^13^ therefore, imaging probes that are capable of detecting soluble forms are highly desirable for early diagnosis. However, strategies that can be utilized to selectively detect the soluble forms are still in short supply.

There are multiple obstacles that need to be overcome in designing small molecules to differentiate between the soluble and insoluble forms of amyloids. First, rare crystal structures of amyloids are available for conventional computer aided design (CAD). Secondly, the basic units of the soluble and insoluble amyloids are the same peptides or proteins. Thirdly, prior knowledge about what the design principles are is rare.^14^–^16^ In this report, we describe a stereo-hindrance tuning strategy for designing probes to selectively detect soluble Aβ (sAβ) species. We believe that this strategy can be applied to other amyloids as well.

For Aβs, the soluble species include monomers, dimers, and oligomers and the insoluble species include fibrils/aggregates and plaques.^17^–^19^ Initially, it was thought that insoluble deposits/plaques in an AD brain cause neurodegeneration. However, mounting evidence indicates that soluble dimeric and oligomeric Aβs are more neurotoxic than insoluble deposits.^12^,^13^,^20^ It has been shown that amyloid plaque burden has poor correlation with Alzheimer’s disease severity.^12^,^13^,^20^ Nonetheless, in the past few decades, insoluble Aβ (insAβ) plaques have been the primary target for AD drug design and clinical trials, and this could partially explain the failures of AD drug development. On the other hand, the failures suggest that treatment intervention at an early stage is one of the likely reasons for successful AD drug development and recent clinical evidence from an experimental drug provides a strong endorsement for early intervention. Liu-Seifert *et al.* reported that the experimental antibody drug Solanezumab could probably be beneficial for AD patients in their early disease stages.^21^

sAβ species are believed to be the dominant biomarkers in early/presymptomatic stages of AD,^8^–^13^ therefore, imaging probes that are capable of selectively detecting sAβ are highly desirable. In addition, it is well documented that the concentration of insAβ increases with the progression of AD, however, it is not clear whether the concentration of the sAβ species increases or decreases during the progression. To answer this fundamental question, highly selective imaging probes for the sAβ species are also highly desirable.

Numerous fluorescence imaging probes for Aβ species have been reported in the past few decades and most of the probes are responsive to insAβ species.^2^,^22^–^32^ We have previously reported that curcumin-based near infrared fluorescent (NIRF) imaging probes are capable of detecting both soluble and insoluble Aβs;^33^–^36^ nonetheless, there are still very few imaging probes with high selectivity towards sAβ. In this report, we demonstrate that, *via* tuning the stereo-hindrance of the curcumin scaffold, it is feasible to develop NIRF imaging probes for the selective imaging of sAβ species.

## Results and discussions

### 1. Tuning the hindrance to match the accessibility of Aβs

**Fig. 1:**
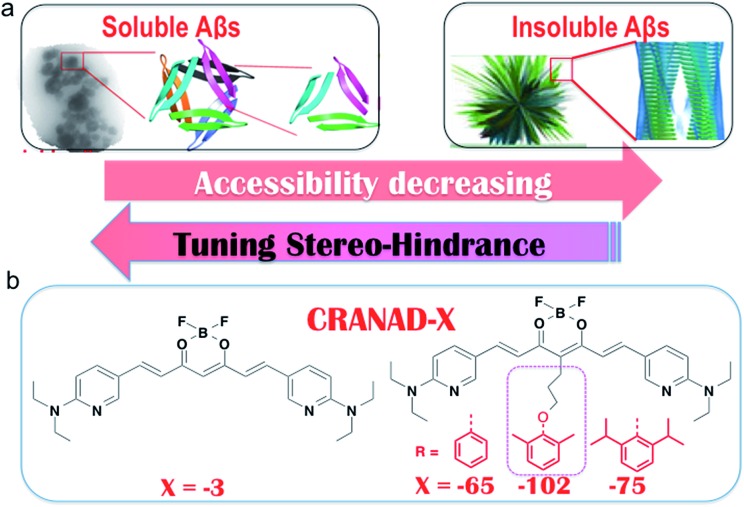
The rationale for designing probes that can selectively detect soluble Aβs. (a) Cartoon pictures suggest that the accessibility decreases from soluble Aβs to insoluble Aβs. (b) The designed imaging probes CRANAD-*X* for tuning the stereo-hindrance to match the accessibility of the Aβ species. We hypothesized that the compact tightness of the Aβs could be harnessed to design small molecules to match their accessibility and the planar moiety of the curcumin scaffold could be inserted into the beta sheets, while the bulky group (red color) could not enter the beta-sheets.

Differentiating between soluble and insoluble Aβs with a small molecular probe is extremely challenging because the basic units of both soluble and insoluble Aβs are essentially the same peptides. It is well known that several anti-Aβ antibodies show certain selectivity towards soluble and insoluble Aβs and that their differentiating capability originates from the differences in the secondary/tertiary structures of the Aβs.^37^–^40^ This provides an indication that the differences between the secondary/tertiary structures can be the basis for designing small molecular probes to differentiate between soluble and insoluble Aβs. Moreover, we noticed that the compactness/accessibility of insoluble and soluble Aβs are significantly different. In addition, our previous molecular docking modeling suggested that a planar curcumin scaffold could intercalate into the beta-sheet of the Aβ peptides and show a significant fluorescence intensity increase upon interacting with both sAβ and insAβ.^24^,^33^–^36^ We reasoned that non-planar “T-Shape” curcumin analogues could have the ability to differentiate between sAβ and insAβ because their compactness and accessibility are different (Fig. 1a). With these considerations in mind, we designed curcumin analogues that contain a planar moiety for intercalating into the beta-sheet and a bulky moiety (T-Shape) to match the tightness and thus to adjust the probe’s accessibility to different Aβs (Fig. 1a). Based on our previously published CRANAD-3, we have designed **CRANAD-65**, **-75**, and **-102** (Fig. 1b) to tune the stereo-hindrance, whose order is **CRANAD-75** > **CRANAD-102** > **CRANAD-65** > **CRANAD-3**. With this series of compounds, we tested their selectivity for sAβ over insAβ species.

**Fig. 2:**
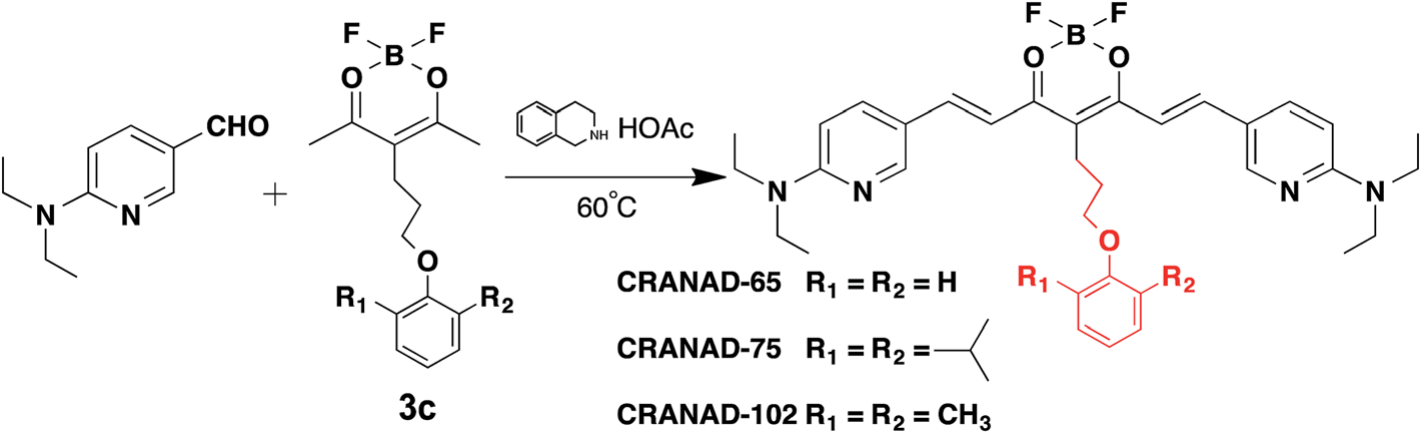
The synthetic route and chemical structures of **CRANAD-65**, **-75**, and **-102**, which have different stereo-hindrance at the 4-position of the curcumin scaffold. The full routes are shown in Fig. S1 in the ESI.†

The synthetic route of the designed compounds is shown in Fig. 2 (more details can be found in the ESI in Fig. S1†) and the synthesis is similar to our previously reported procedures.^33^–^36^ We also recorded their emission spectra under the same excitation conditions and found that **CRANAD-75** had a higher fluorescence intensity than **CRANAD-65** and **-102** (ESI Fig. S2†). This is consistent with the quantum yields of **CRANAD-65**, **-75**, and **-102**, which were 0.03, 0.04, and 0.018 respectively (Cy5.5 was used as the reference). The introduction of a phenoxy-alkyl chain into the curcumin scaffold likely increases the hydrophobicity of the probe, which may lead to strong non-specific binding to blood serum. To investigate whether the probes have a strong interaction with serum, we incubated the probes with different concentrations of bovine serum albumin (BSA) and found that there were no apparent changes in the fluorescence properties (ESI Fig. S3†), suggesting serum has minimal interference in the fluorescence of the probes. In addition, we have investigated the cytotoxicity of these probes and found that there was no significant toxicity (5.0 μM of probes were used) (ESI Fig. S4†).

### 2. **CRANAD-102** is selective for soluble Aβs, but not for insoluble Aβs

**Fig. 3:**
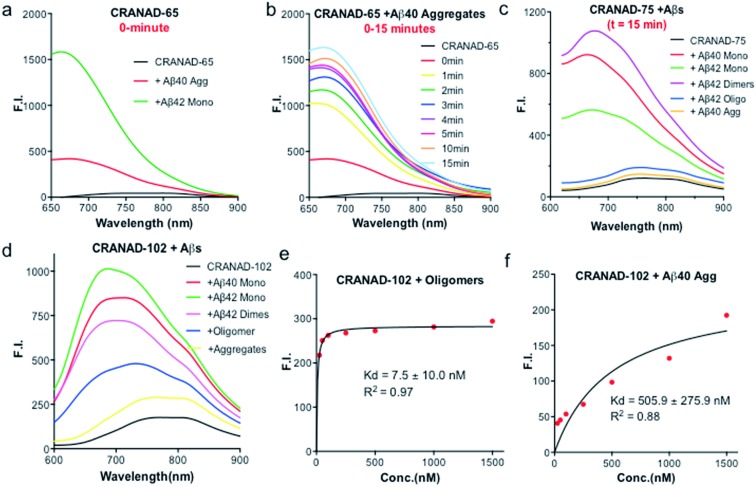
(a) *In vitro* fluorescence testing of **CRANAD-65** (250 nM) with insoluble aggregates (red) and soluble monomers (green) at *t* = 0 min (Ex = 580 nm). (b) Fluorescence intensity change of **CRANAD-65** with aggregates at different times (Ex = 580 nm). (c and d) Fluorescence testing of **CRANAD-75** and **CRANAD-102** with insoluble aggregates and soluble monomers at *t* = 15 min (Ex = 580 nm). (e and f) *K*_d_ measurements of **CRANAD-102** with oligomers and aggregates.

Our previous data showed that CRANAD-3 had excellent responses to both sAβ and insAβs.^33^–^36^ To investigate whether the introduction of the phenyloxy alkyl chain at the 4-position of the curcumin scaffold can lead to certain selectivity for sAβ over insAβ, we incubated **CRANAD-65** with monomeric Aβs and aggregated Aβs. We found that **CRANAD-65** showed considerable selectivity at the very beginning of the incubation (*t* = 0 min, Fig. 3a); however, the selectivity decreased rapidly with the increase in incubation time (*t* = 0–15 min, Fig. 3b), which was most likely due to the limited hindrance of the phenyl ring at the 4-position. **CRANAD-65** also showed apparent responses to Aβ40 dimers and Aβ42 oligomers (ESI Fig. S5†). To increase the hindrance, we tested **CRANAD-75** with monomeric Aβs and aggregated Aβs and we found that **CRANAD-75** showed excellent selectivity for monomeric Aβs. At 680 nm, the fluorescence intensity of **CRANAD-75** with monomeric Aβ42 was about 88.5-fold higher than that with insoluble Aβ40 aggregates. Unlike **CRANAD-65**, the selectivity did not change with an increase in incubation time (Fig. 3c), which strongly suggests that the two isopropyl groups in **CRANAD-75** significantly contribute to the consistent selectivity. However, no significant fluorescence intensity increase was observed when **CRANAD-75** was incubated with oligomeric Aβs, suggesting that **CRANAD-75** had a poor response to soluble oligomeric Aβs. This also indicated that the hindrance from the two isopropyl groups on the phenyl ring was too bulky to prevent it from sliding into the beta sheets of the oligomers. To investigate whether decreasing the hindrance can increase the accessibility of oligomeric Aβs, **CRANAD-102**, in which two methyl groups were used to replace the two isopropyl groups in **CRANAD-75**, was incubated with oligomeric Aβs. We found that **CRANAD-102** could reliably respond to oligomeric Aβs and a 4.33-fold fluorescence intensity increase at 700 nm was observed (Fig. 3d).

We further measured the binding affinity of **CRANAD-102** for soluble and insoluble Aβs. In order to measure the *K*_d_ values of **CRANAD-102** for soluble and insoluble Aβs, we titrated 750 nM of Aβ with different concentrations of **CRANAD-102**. Remarkably, we found that the *K*_d_ value for sAβ was 68-fold higher than that for insAβ (7.5 ± 10 nM *vs.* 505.9 ± 275.9 nM) (Fig. 3e and f). Again, these data strongly indicate that **CRANAD-102** is highly selective for sAβ (ESI Fig. S6†). Our data also clearly suggested that tuning the hindrance of small molecules could match the tightness of different Aβs and thus enable us to differentiate between sAβ and insAβ species. In addition, we found that **CRANAD-102** had good specificity for sAβ species over other aggregation-prone peptides/proteins, including tau, α-synuclein, and amylin (ESI Fig. S7†).

### 3. Brain phantom imaging of soluble and insoluble Aβs with **CRANAD-102**

**Fig. 4:**
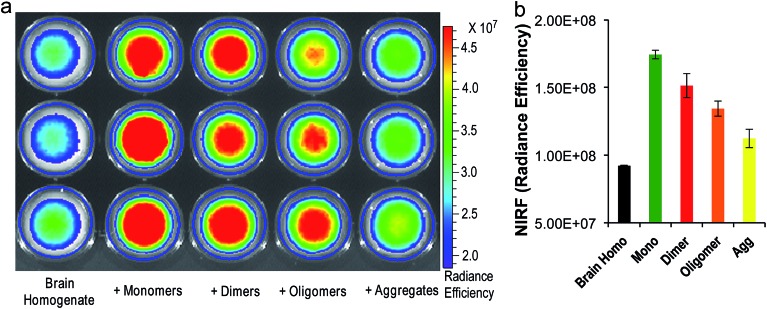
(a) Phantom imaging of brain homogenates with **CRANAD-102** in the presence of different Aβs (triplicates). (b) Quantitative analysis of the images in (a).

To investigate whether **CRANAD-102** can differentiate between soluble and insoluble Aβs in a relevant biological environment, we used phantom imaging with soluble Aβ40 monomers, Aβ40 dimers, Aβ42 oligomers, and insoluble Aβ40 aggregates. In this experiment, we added the same amount of Aβ species to mouse brain homogenates and then imaged the brain homogenates after the addition of **CRANAD-102**. Fig. 4a and b clearly show that the fluorescence intensities from the homogenates with the Aβ40 monomers and Aβ42 oligomers were much higher than those with the insoluble Aβ40 aggregates, indicating that **CRANAD-102** had significant selectivity for sAβ over insAβ in brain-relevant biological environments.

### 4. Histological staining of a brain slice with **CRANAD-102**

To investigate whether **CRANAD-102** can stain insoluble Aβ plaques in brain slices, we incubated it with a brain slice from a 14 month old APP/PS1 mouse. No apparent plaque staining was observed (ESI Fig. S8†), suggesting that **CRANAD-102** is not sensitive to insoluble Aβ species. However, it is impossible to use **CRANAD-102** to visualize soluble Aβs because the size of the sAβ species is too small for observation under a fluorescence microscope. Notably, our results are different to previous reports, in which Aβ deposits/plaques could be visualized with imaging probes that have claimed to be selective for soluble Aβ oligomers.^14^,^15^

### 5. **CRANAD-102** has excellent blood–brain barrier penetration

Decent blood–brain barrier (BBB) penetration is a crucial requirement for a brain-imaging probe. To validate whether **CRANAD-102** can cross the BBB, we injected it into wild type mice. After 1 hour, the mice were sacrificed and their brains were dissected. We used ethyl acetate to extract **CRANAD-102** from the brain homogenates and found that both the fluorescence spectra and LC-MS data confirmed the existence of **CRANAD-102** in the extraction (ESI Fig. S9†), suggesting that **CRANAD-102** could cross the BBB and thus could be an excellent brain imaging probe. Before *in vivo* imaging, we also investigated the stability of **CRANAD-102** in serum, which can provide an indication of the potential stability of the probe in blood. In this regard, we incubated **CRANAD-102** in mouse serum at 37 °C for 0, 30, and 60 min and measured its stability using HPLC and found that nearly 80% of **CRANAD-102** remained after 60 min incubation (ESI Fig. S10†), suggesting that it could have certain stability in blood.

### 6. *In vivo* NIRF imaging and the monitoring of sAβ concentration changes with **CRANAD-102**

**Fig. 5:**
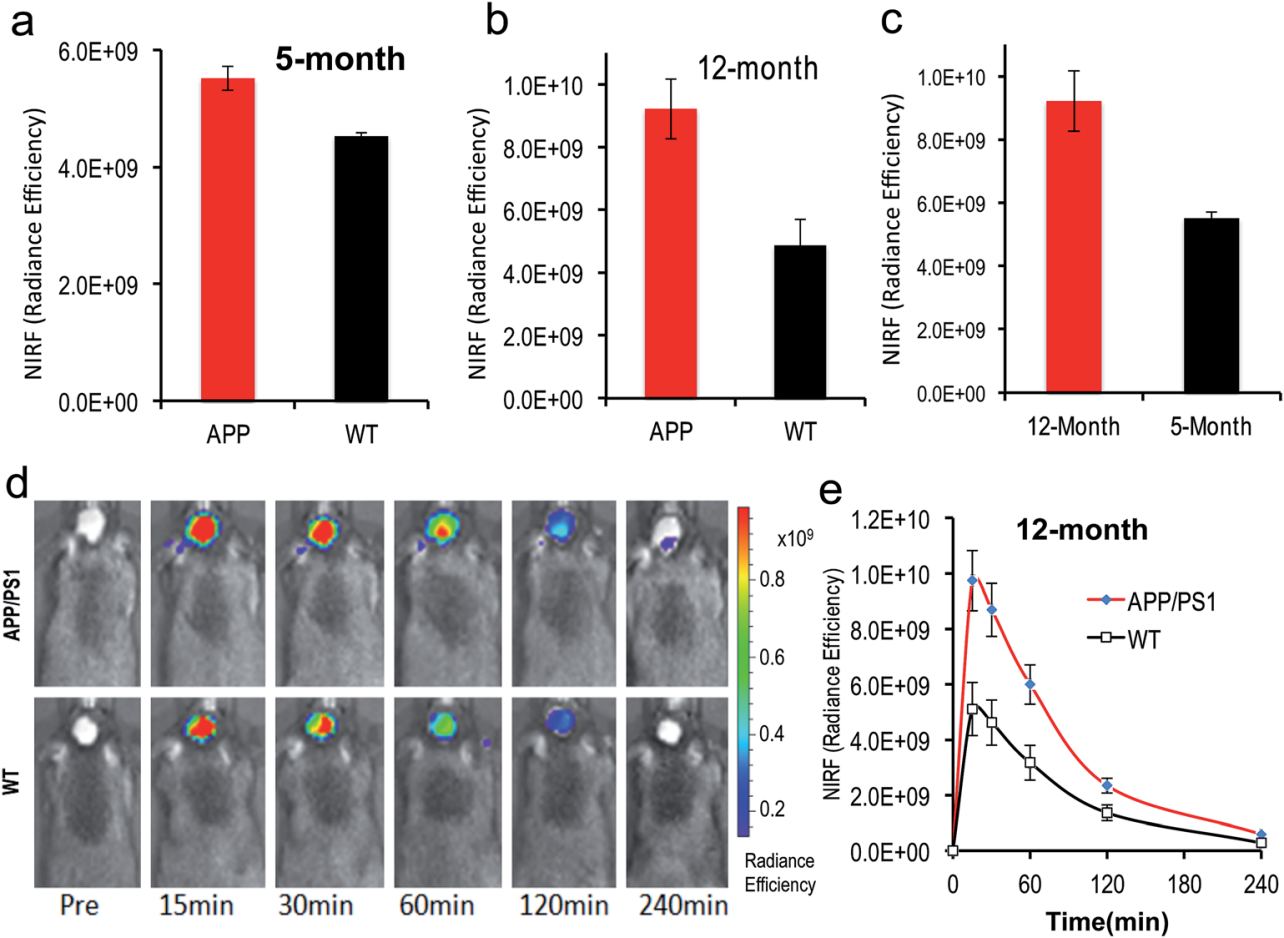
*In vivo* NIRF imaging with **CRANAD-102** to detect the differences between AD mice and WT mice, and AD mice at different ages. (a) The NIRF signal of **CRANAD-102** from 5 month old APP/PS1 mice and WT mice at 30 minutes after IV injection; (b) NIRF signal of **CRANAD-102** from 12 month old APP/PS1 mice and WT mice at 30 minutes after IV injection; (c) NIRF signal of **CRANAD-102** from 5 month and 14 month old APP/PS1 mice at 30 minutes after IV injection; (d) representative images of the *in vivo* imaging of 14 month old APP/PS1 and WT mice at 0, 15, 30, 60, 120, and 240 minutes after IV injection of **CRANAD-102**. (e) Time-course curves of NIRF from **CRANAD-102** in APP/PS1 and WT mice.

To test whether **CRANAD-102** was able to detect soluble species *in vivo* by NIRF imaging, we first tested it with 4 month old APP/PS1 mice. At this age sAβ species are the predominant species in the brain.^41^–^43^ After intravenous injection, we found that the fluorescence signals from the brains of APP/PS1 mice were higher (1.22-fold at 30 min after injection) than those from the age-matched wild type (WT) mice (Fig. 5a), suggesting that **CRANAD-102** was indeed capable of detecting soluble Aβ species *in vivo*.

To investigate whether **CRANAD-102** can be used to detect the changes in sAβ concentration with the progression of AD, we performed NIRF imaging with 12 month old APP/PS1 mice. Our data indicated that the difference between APP/PS1 mice and age-matched WT mice was about 1.89-fold at 30 min after the injection (Fig. 5b, d and e), suggesting that **CRANAD-102** could be used to detect the concentration changes of sAβ at different ages and the increase of sAβ was about 1.54-fold from the age of 5 months old to 12 months old (Fig. 5c). This result suggested that, like the insAβ species, the concentration of sAβ also increased with ageing and the progression of AD.

## Conclusion

In summary, our results suggest that the stereo-hindrance tuning strategy is feasible for designing imaging probes to differentiate between soluble and insoluble Aβs. With **CRANAD-102**, we demonstrated that monitoring the changes in sAβ concentration during the progression of AD was achievable. We believe that our imaging technology will greatly assist AD drug discovery that is aimed at the sAβ-dominated pre-symptomatic stage. Our hindrance tuning strategy is unique, because it is different from traditional SAR-based compound design. Our results also suggest that the tightness/accessibility of amyloids can be considered to be the basis for differentiating between soluble and insoluble forms and we believe that our strategy has paved a way for designing imaging probes for different forms of amyloids.

## Materials and methods

The reagents used for the synthesis were purchased from Aldrich and used without further purification. Column chromatography was performed on silica gel (SiliCycle Inc., 60 Å, 40–63 mm) slurry packed into glass columns. Synthetic Aβ peptide (1–40/42) is from rPeptide (Bogart, GA, 30622). Aβ dimers of S26C Aβ40 were purchased from AnaSpec (Fremont, CA, 94555). **CRANAD-65**, **-75** and **-102** were dissolved in DMSO to prepare a 25.0 μM stock solution. ^1^H and ^13^C NMR spectra were recorded at 500 MHz and 125 MHz respectively, and reported in ppm downfield from tetramethylsilane. Fluorescence measurements were carried out using an F-4500 Fluorescence Spectrophotometer (Hitachi). Mass spectra were obtained at the Department of Pharmaceutical Analysis of China Pharmaceutical University. Transgenic female APP-PS1 mice and age-matched wild type female mice were purchased from Jackson Laboratory. All animal experiments were performed in compliance with institutional guidelines and were approved by the IACUC Committee at Massachusetts General Hospital.

### 1. Synthesis of **CRANAD-102**

The synthesis of compound **3c** was performed according to a modified protocol of our previously reported procedure.^33^**3c** (100 mg, 0.32 mmol) was dissolved in acetonitrile (3.0 mL), followed by the addition of acetic acid (6.7 μL, 0.1 mmol), tetrahydroisoquinoline (9.3 μL, 0.1 mmol), and 6-(diethylamino) nicotinaldehyde (173 mg, 0.96 mmol). The resulting solution was stirred at room temperature for 4 h. A black residue, which was obtained after removing the solvent, was subjected to flash column chromatography with methylene chloride to give a black powder (**CRANAD-102**, 180 mg, 88%). m.p.: 210–215 °C. ^1^H NMR (300 MHz, CDCl_3_) *δ* (ppm): 8.35 (d, 2H, *J* = 2.13 Hz, 2ArH), 8.03 (d, 2H, *J* = 14.97 Hz, 2 –CH=CH–), 7.68 (dd, 2H, *J* = 2.13, 9.18 Hz, 2ArH), 7.06 (m, 2H, 2 ArH), 6.96 (m, 3H, 2 –CH=CH–), 6.40 (d, 2H, *J* = 9.18 Hz, 2ArH), 3.89 (t, 2H, *J* = 5.29 Hz, –O–CH_2_–CH_2_–), 3.55 (q, 8H, *J* = 7.05 Hz, 2 –N(CH_2_CH_3_)CH_2_CH_3_), 2.89 (t, 2H, *J* = 7.5 Hz, –O–CH_2_–CH_2_–CH_2_–), 2.31 (s, 6H, 2 Ar–CH_3_), 2.03 (m, 2H, –O–CH_2_–CH_2_–CH_2_–), 1.20 (t, 12H, *J* = 7.05 Hz, 2 –N(CH_2_CH_3_)CH_2_CH_3_). ESI-MS (M + H) *m*/*z*: 631.4. HRMS calculated for C_36_H_46_BF_2_N_4_O_3_, *m*/*z*, 630.3662 [(M + H)]^+^; found, 630.3662. ^13^C NMR (CDCl_3_) *δ* (ppm): 12.92, 16.47, 22.31, 32.16, 43.06, 70.93, 106.82, 110.27, 112.26, 118.84, 123.87, 128.92, 130.93, 135.56, 145.09, 153.02, 155.93, 158.57, 176.49. HPLC purity: 98.83%.

The details of the synthesis of **CRANAD-65**, **-75** and **-102** can be found in the ESI.†

### 2. Quantum yield measurement for **CRANAD-65**, **-75**, and **-102**

The measurements were conducted by following the reported protocol,^44^ and Cy5.5 was used as the reference.

### 3. MTT cytotoxicity of **CRANAD-65**, **-75**, and **-102**

HEK293 cell lines were seeded at 5 × 10^3^ cells per well in 96-well microliter plates. After 24 h, cells were exposed to **CRANAD-65** (5.0 μM) for 48 hours. Then, cell survival was determined by the addition of an MTT solution (20 μL of 5 mg mL^–1^ MTT in PBS). After 6 h, the medium was removed by aspiration. The cells were dissolved in 150 mL DMSO and optical absorbance was measured at 570 nm using a SpectraMax M2 Microplate Reader (Molecular Devices). The survival ratios were expressed in percentages with respect to the untreated cells.

### 4. Preparation of the Aβ40/42 monomers, Aβ42 oligomers and Aβ40 aggregates

The preparations were performed according to previous reports.^35^,^36^,^38^

### 5. Fluorescence spectral testing of CRANAD-*X* (*X* = 65, 75, and 102) with Aβs

To test the interactions of CRANAD-*X* with Aβs, the following procedure was followed. Step 1: 1.0 mL of PBS buffer was added to a quartz cuvette as a blank control and its fluorescence was recorded using the same parameters as for CRANAD-*X*. Step 2: the fluorescence of a CRANAD-*X* solution (1.0 mL, 250 nM) was recorded with excitation at 610 nm and emission from 630 nm to 900 nm. Step 3: to the above CRANAD-*X* solution, 30 μL of Aβs (25 μM stock solution in HFIP for the monomers and dimers, and 25 μM stock solution in PBS buffer or double distilled water for the oligomers and Aβ40 aggregates) was added to make a final Aβ concentration of 750 nM. The fluorescence readings from this solution were recorded as described in step 2. The final spectra from steps 2 and 3 were corrected using the blank control from step 1.

### 6. Brain phantom imaging

A 4 month old wild type (B6C3F1/J) mouse was sacrificed and its brain was dissected. The brain was then homogenized with 2.0 mL of PBS and 0.1 mL of homogenate was added to 15 wells of a 96-well plate, followed by the addition of **CRANAD-102** (2.5 μM, 10 μL) and the Aβ monomers, oligomers, and aggregates (2.5 μM, 30 μL). The resulting brain homogenates were imaged with Ex/Em = 640/700 nm using an IVIS®Spectrum imaging system.

### 7. Stability of **CRANAD-102** in serum

**CRANAD-102** (50 μL, 10% DMSO solution, 10 μM) was incubated with 300 μL of mouse serum at 37 °C for 0, 30, and 60 min. The proteins in the serum were precipitated by adding 500 μL of acetonitrile after centrifugation at 5000 rpm for 15 min at 4 °C, and the supernatant was collected. Approximately 0.1 mL of the supernatant solution was analyzed using HPLC (UV detector, *λ* = 420 nm).

### 8. *In vivo* NIRF imaging

*In vivo* NIRF imaging was performed using an IVIS®Spectrum animal imaging system (Caliper LifeSciences, Perkin Elmer, Hopkinton, MA). The images were acquired with a 640 nm excitation filter and a 700 nm emission filter. Data analysis was performed using LivingImage® 4.2.1 software.

Five-month old mice (female transgenic APP-PS1, *n* = 3–4 and age-matched female wild type control mice, *n* = 3–4) were shaved before background imaging. An injection solution of **CRANAD-102** (2 mg kg^–1^) was freshly prepared in 15% DMSO, 15% cremorphor, and 70% PBS and the solution was stabilized for 20 min before injection. Each mouse was injected intravenously with 100 μL of **CRANAD-102**. Fluorescence signals from the brain were recorded before and 15, 30, 60, 120, and 240 min after intravenous injection of the probe. To evaluate our imaging results, an ROI was drawn around the brain region. The *P* values were calculated using Student’s *t*-test.

### 9. Histological staining of brain slices

A 30-micron brain slice from a 14 month old APP/PS1 mouse was incubated with 1% **CRANAD-102** solution (20% ethanol and 80% dd water) for 15 minutes and then washed with 20% ethanol followed by washing with dd water. The slice was covered with VectaShield mounting media. A consecutive slide was stained with 1% Thioflavin S solution (20% ethanol and 80% dd water). The fluorescence images were taken using a Nikon Eclipse 50i microscope.

## Conflicts of interest

There are no conflicts to declare.

## Supplementary Material

Supplementary information
