# Supplementary material for: Tuning the stereo-hindrance of a curcumin scaffold for the selective imaging of the soluble forms of amyloid beta species
Source: Chem Sci. 2017 Sep 15;8(11):7710–7. doi: 10.1039/c7sc02050c (PMC5851342; doi:10.1039/c7sc02050c)
Supplement: Supplementary file 1 [file SC-008-C7SC02050C-s001.pdf]

Electronic Supplementary Material (ESI) for Chemical Science.
This journal is © The Royal Society of Chemistry 2017

Tuning Stereo-hindrance of Curcumin Scaffold for Selective Imaging of Soluble Forms of Amyloid Beta Species

Yuyan Li [1,2,5], Jian Yang[1,2,5], Hongwu Liu[2], Jing Yang[1,3], Lei Du[2], Haiwei Feng[2], Yanli Tian[1,4], Jianqin Cao[2], and Chongzhao Ran[1 *]

[1]Molecular Imaging Laboratory, MGH/MIT/HMS Athinoula A. Martinos Center for Biomedical Imaging, Department of Radiology, Massachusetts General Hospital/Harvard Medical School, Charlestown, Massachusetts 02129; [2]School of Pharmacy, China Pharmaceutical University, Nanjing, China; 210009; [3]College of Pharmaceutical Sciences, Soochow University, Suzhou, China; 215006; [4]Department of Parasitology, Zhongshan School of Medicine, Sun Yat-Sen University, Guangzhou, P. R. China; [5]These authors contributed equally to this work.

Contents

1. Materials and Methods……………………………………………………………Page 3-7

Synthetic route (SI Fig.1)…………………………………………………………..Page 3

Synthesis of CRANAD-65…………………………………………………………Page 3

Synthesis of CRANAD-75…………………………………………………………Page 5

Synthesis of CRANAD-102………………………………………………………..Page 5

2. Supplemental Figure 1-4 …………………………………………………………Page 7-8

SI Fig.1………………………………………………………………………………...Page 3

SI Fig.2………………………………………………………………………………...Page 7

SI Fig.3………………………………………………………………………………...Page 7

SI Fig.4………………………………………………………………………………...Page 8

SI Fig.5………………………………………………………………………………...Page 8

SI Fig.6………………………………………………………………………………...Page 9

SI Fig.7………………………………………………………………………………...Page 9

SI Fig.8………………………………………………………………………………...Page 10

SI Fig.9………………………………………………………………………………...Page 11

SI Fig.10………………………………………………………………………………Page 11

3. ^{1}H NMR, ^{13}C NMR, HR-MS，HPLC …………………………………………Page 12-17

1, Materials and Methods

Reagents used for the synthesis were purchased from Aldrich and used without further purification. Column chromatography was performed on silica gel (SiliCycle Inc., 60 Å, 40-63 mm) slurry packed into glass columns. Synthetic Aβ peptide (1-40/42) is from rPeptide (Bogart, GA, 30622). Aβ dimers of S26C Aβ40 were purchased from AnaSpec (Fremont, CA, 94555). CRANAD-65, -75 and -102 were dissolved in DMSO to prepare a 25.0 μM stock solution. ^{1}H and ^{13}C NMR spectra were recorded at 500 MHz and 125 MHz respectively, and reported in ppm downfield from tetramethylsilane. HPLC was run on a Shimadzu LC-20A machine; mobile phase: 30% water in CH_3CN containing 0.1% TFA, UV detection at 420 nm. Fluorescence measurements were carried out using an F-4500 Fluorescence Spectrophotometer (Hitachi). Mass spectra were obtained at the Department of Pharmaceutical Analysis of China Pharmaceutical University. Transgenic female APP-PS1 mice and age-matched wild type female mice were purchased from Jackson Laboratory. All animal experiments were performed in compliance with institutional guidelines and were approved by the IACUC Committee at Massachusetts General Hospital.

Synthesis of CRNAD-65, -75 and -102.

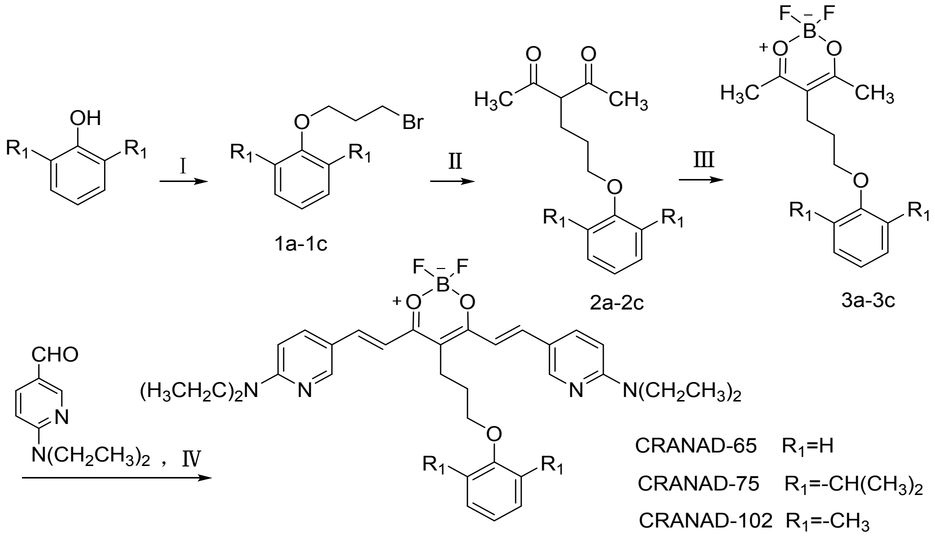

SI Fig.1 Reagents and conditions: (I) 1,3-dibromoprone, NaOH, H_2O, reflux, r.t.; (II) 2,4-petanedione, dry K_2CO_3, anhydrous acetone, reflux; (III) BF_3•$(CH_3CH_2)_2O$, tributyl borate, r.t.; (IV) tetrahydroisoquinoline, acetic acid, CH_3CN, r.t.

The syntheses of CRANAD-65, CRANAD-75 and CRANAD-102 were shown in **SI Fig.1** and their structures were confirmed by NMR and ESI-MS spectra.

Synthesis of CRANAD-65

(I) Synthesis of 1a. Phenol (2g, 21.2 mmol) was slowly added to NaOH (1.77g, 44.3 mmol) in DMF (5mL), and the reaction mixture was then stirred at 0°C for 2 h on an ice bath under protection of nitrogen gas. The supernatant of reaction mixture was then added to a mixture of 1, 3-dibromopropane (3.93mL, 37.7 mmol) and K_2CO_3 (5.42 g, 39.3 mmol), and the resulting mixture was stirred at r.t. for 6 h. The reaction mixture was poured in water, and then extracted with ethyl acetate, washed with saturated NaCl, and dried over $MgSO_4$. 1-(3-bromopropoxy)-benzene (1a, 45.0% yield) was obtained as oily liquid after flash column chromatography (Petroleum ether as elute). ^{1}H NMR (300MHz $CDCl_3$) δ (ppm): 7.29-7.23 (m, 2H), 6.97-6.87 (m, 3H), 4.06-4.02 (t, 2H), 3.67-3.64 (t, 2H), 3.37-3.32 (m, 2H).

(II) Synthesis of 2a. 1-(3-bromopropoxy)-benzene (1a, 1 g, 4.7 mmol) was dissolved in acetone (10 mL), and then K_2CO_3 (860 mg, 6.2 mmol), KI (c.a.) and acetylacetone (473µL, 4.4 mmol) were added to the mixture, which were stirred at 65°C for 20h. The reaction mixture was poured in water, and then extracted with ethyl acetate (3×15 mL), then washed with saturated NaCl, and dried over Na_2SO_4. A yellowish oily liquid of 2a was obtained as pale yellow oily liquid after flash column chromatography (10:1 Petroleum ether-ethyl acetate) (51.8% yield).

^{1}H NMR (300 MHz, $CDCl_3$) δ: 16.69 (s), 7.25-7.14 (m, 2H), 6.91-6.75 (m, 3H), 3.88 (t, *J* = 10.1, 4.9 Hz, 2H), 3.64 (t, *J* = 7.2 Hz, 1H), 2.41-2.33 (m, 2H), 2.09 (d, *J* = 12.4 Hz, 6H), 2.01-1.91 (m, 2H), 1.86-1.74 (m, 2H).

(Ⅲ) Synthesis of 3a. $BF_3 \cdot Et_2O$ was slowly added to 3-(3-phenoxy-propyl) pentane-2, 4-dione (2a, 800 mg, 3.05mmol) in tributyl borate (768uL, 6.10mmol) under the protection of nitrogen. The reaction mixture was then stirred at r.t. for 4 h. A white crystal (91.0% yield) was obtained after removing the solvent. ^{1}H NMR ($CDCl_3$) δ (ppm): 1.92(m, 2H), 2.36(s, 6H), 2.58(t, 2H, *J* = 7.5Hz), 3.99 (t, 2H, *J* = 5.5Hz,), 6.89 (d, 2H, *J* = 9Hz), 6.97 (t, 1H, *J* = 6Hz), 7.30 (m, 2H).

(Ⅳ)CRANAD-65 3-(3-phenoxy-propyl)-2’,2’-difluoro-1,3-dioxaboryl-pentane-2,4-dione (100 mg, 0.36mmol) was dissolved in acetonitrile (3.0 mL), followed by the additions of acetic acid (6.7ul, 0.1mmol), tetrahydroisoquinoline (9.3ul, 0.1mmol), and 6-(diethylamino) nicotinaldehyde (173 mg, 0.96mmol). The resulting solution was stirred at r.t. for 4h. A black residue obtained after removing the solvent was subjected to flash column chromatography with methylene chloride to give a black powder (45.6% yield). m.p.: 202-205°C. ^{1}H NMR (300 MHz, $CDCl_3$) δ (ppm): 8.32 (d, 2H, *J* = 2.0 Hz, ArH), 7.99 (d, 2H, *J* = 15.0 Hz, ArH), 7.56 (dd, 2H, *J* = 9.1, 2.1 Hz, ArH), 7.32-7.22 (m, 2H, ArH), 6.95 (d, 3H, *J*=8.0 Hz, ArH), 6.84 (d, 2H, *J* =15.0Hz, -CH=CH-), 6.41 (d, 2H, J=9.1 Hz, -CH=CH-), 4.04 (t, 2H, *J* = 5.2 Hz, $-OCH_2-$), 3.57 (q, 8H, *J*=7.0 Hz, N-$\underline{CH_2}CH_3$), 2.78 (t, 2H, *J* = 7.1 Hz, $-CH_2CH_2\underline{CH_2}-$), 2.12-1.95(m, 2H,

-CH2<u>CH2</u>CH2-), 1.22 (t, *J* = 7.0Hz, 12H, N-CH2<u>CH3</u>). ESI-MS $(M+Na)^+$ m/z: 624.4. HRMS calculated for $C_{34}H_{42}BF_2N_4O_3$, m/z, 602.3349 $[(M + H)]^+$; found, 602.3352. ^{13}C NMR (75 MHz, $CDCl_3$) δ (ppm): 176.10, 158.13, 152.55, 144.77, 135.01, 129.06, 120.34, 118.24, 114.04, 111.56, 109.12, 105.55, 76.96, 76.54, 76.11, 65.30, 42.59, 30.17, 21.10, 12.45. HPLC Purity：98.720%.

Synthesis of CRANAD-75

(I) Synthesis of 1b. The similar protocol described above for preparing compound 1a was used, except phenol was replaced by 2,6-diisopropylphenol, and an oily liquid of 1b was obtained (39.0%). ^1H NMR (300 MHz, DMSO) δ (ppm): 7.15-7.04 (m, 3H), 3.85-3.82(t, 2H), 3.54-3.48 (m, 8H), 3.28-3.32 (m, 2H), 2.85-2.81 (t, 2H), 1.17-1.13 (d, *J* = 12 Hz, 12H).

(Ⅱ) Synthesis of 2b. The similar protocol described above for preparing compound 2a was used, and a colourless oily liquid of 2b was obtained (80.0% yield). ^1H NMR (300MHz, CDCl3) δ (ppm): 16.83 (s, 1H), 7.14-7.03 (m, 3H), 3.96-3.84 (m, 1H), 3.72 (t, *J* = 6.3 Hz, 1H), 3.66 (t, *J* = 6.3 Hz, 1H), 3.24 (dq, *J* = 13.6, 6.8 Hz, 2H), 2.17 (t, *J* = 7.1 Hz, 5H), 2.08 (s, 1H), 1.66-1.46 (m, 1H), 1.41 (dd, *J* = 15.7, 8.6 Hz, 1H), 1.17 (dd, *J* = 6.9, 2.2 Hz, 12H).

(Ⅲ) Synthesis of 3b. The similar protocol described above for preparing compound 3a was used, and a white crystal of 3a was obtained (83.0% yield). ^1H NMR (300 MHz, $CDCl_3$) δ (ppm): 7.10 (d, *J*= 2.0Hz, 3H), 3.76(t, *J* = 6.2, 4.3 Hz, 2H), 3.28 (t, *J*=6.9 Hz, 2H), 2.48-2.39 (m, 2H), 2.39 (s, 3H,), 1.76-1.63 (m, 2H), 1.23 (d, *J* = 6.9Hz, 15H)

(IV) CRANAND-75. The similar protocol described above for preparing CRANAD-65 was used, and a deep green fine powder of CRANAD-75 was obtained (47.0% yield) m.p.:185-188°C. ^1H NMR (300MHz, $CDCl_3$) *δ*: 8.32-8.31 (d, 2H, *J*= 3Hz, 2ArH), 8.01-7.96 (d, 2H, *J*= 15Hz, 2 –CH=CH-), 7.65-7.62 (dd, 2H, *J*= 1.98Hz, *J*= 1.80Hz, ArH), 7.11 (s, 3H, 3ArH)), 6.94-6.90 (d, 2H, *J*= 12 Hz, 2 –CH=CH-)，6.36-6.33 (d, 2H, *J*= 9 Hz, 2ArH), 3.85-3.82 (t, 2H, *J*= 5.29 Hz, -O-<u>CH2</u>CH2-), 3.55-3.48(q, *J*= 6.84Hz, 8H, N-<u>CH2</u>CH3), 3.32-3.28 (t, 2H, *J*=5.29Hz, -O-CH2 CH2<u>CH2</u>-), 2.85(m, 2H, Ar-<u>CH</u>(CH3)2), 2.00(m, 2H, -O-CH2<u>CH2</u>CH2-), 1.21-1.15(m, 12H, N-CH2<u>CH3</u>), 1.18-1.13(d,12H, m, 2 -CH<u>(CH3)2</u>). ESI-MS (M+H) m/z: 686.3. HRMS calculated for $C_{40}H_{54}BF_2N_4O_3$, m/z, 686.4288 $[(M + H)]^+$; found, 686.4277. ^{13}C NMR (75 MHz, $CDCl_3$) δ (ppm):176.46, 158.54, 153.32, 153.02, 145.10, 141.86, 135.55, 124.64, 124.08, 118.80, 112.16, 110.24, 106.10, 73.65, 43.07, 32.18, 26.69, 24.11, 22.40, 12.91. HPLC Purity: 96.041%.

Synthesis CRANAD-102

(I) Synthesis of 1c. The same reaction described above to prepare compound 1a was used, except phenol was replaced by 2,6-dimethyl-phenol, (1c, 3.02 g, 76%) was obtained as oily liquid after flash column chromatography (Petroleum ether as elute). ^{1}H NMR (300 MHz, $CDCl_3$) δ (ppm): 7.04 (d, 2H, *J*=7.44 Hz), 6.96 (m, 1H), 3.92 (t, 2H, *J*=5.77 Hz), 3.73 (t, 2H, *J*=6.43 Hz), 2.36 (m, 2H), 2.32 (s, 6H).

(Ⅱ) Synthesis of 2c. The similar protocol described above for preparing compound 2a was used. 2c was obtained (433 mg, 40%) as pale yellow oily liquid after flash column chromatography (10:1 Petroleum ether-ethyl acetate). ^{1}H NMR (300 MHz, $CDCl_3$) δ (ppm): 17.10 (s), 7.05 (d, 2H, *J*=7.09 Hz), 6.89 (m, 1H), 3.82 (d, 2H, *J*=5.21 Hz), 3.28 (t, 1H, *J*=4.49 Hz), 2.31 (s, 6H), 2.20 (s, 6H), 1.94 (m, 2H), 1.78 (m, 2H).

(Ⅲ) Synthesis of 3c. The similar protocol described above for preparing compound 3a was used. A white crystal (3c, 785 mg, 83%) was obtained after removing the solvent. ^{1}H NMR (300 MHz, $CDCl_3$) δ (ppm): 6.98 (d, 2H, *J*=7.35 Hz), 6.90 (m, 1H), 3.77 (t, 2H, *J*=5.65 Hz), 2.61 (t, 2H, *J*=5.65 Hz), 2.37 (s, 6H), 2.23 (s, 6H), 1.86 (m, 2H).

(IV) Synthesis of CRANAD-102. 3c (100 mg, 0.32mmol) was dissolved in acetonitrile (3.0 mL), followed by the additions of acetic acid (6.7ul, 0.1mmol), tetrahydroisoquinoline (9.3ul, 0.1mmol), and 6-(diethylamino) nicotinaldehyde (173 mg, 0.96mmol). The resulting solution was stirred at r.t. for 4h. A black residue obtained after removing the solvent was subjected to flash column chromatography with methylene chloride to give a black powder. (180 mg, 88%). m.p.: 210-215°C. ^{1}H NMR (300 MHz, $CDCl_3$) δ (ppm): 8.35 (d, 2H, *J*=2.13 Hz, 2ArH), 8.03 (d, 2H, *J*=14.97 Hz, –CH=CH-), 7.68 (dd, 2H, *J*=2.13, 9.18 Hz, ArH), 7.06 (m, 2H, ArH), 6.96 (m, 3H, 2 –CH=CH-, ArH), 6.40 (d, 2H, *J*=9.18 Hz, ArH), 3.89 (t, 2H, *J*=5.29 Hz, -O-$\underline{CH_2}CH_2$-), 3.55 (q, 8H, *J*=7.05 Hz, N-$\underline{CH_2}CH_3$), 2.89 (t, 2H, *J*=7.5 Hz, -O-$CH_2CH_2\underline{CH_2}$-), 2.31 (s, 6H, 2 Ar-CH_3), 2.03 (m, 2H, -O-$CH_2\underline{CH_2}CH_2$-), 1.20 (t, 12H, *J*=7.05 Hz, 4 -CH_2CH_3). ESI-MS (M+H) m/z: 630.4. HRMS calculated for $C_{36}H_{46}BF_2N_4O_3$, m/z, 630.3662 $[(M + H)]^+$; found, 630.3662. ^{13}C NMR ($CDCl_3$) δ (ppm):12.92, 16.47, 22.31, 32.16, 43.06, 70.93, 106.82, 110.27, 112.26, 118.84, 123.87, 128.92, 130.93, 135.56, 145.09, 153.02, 155.93, 158.57, 176.49. HPLC Purity: 98.828%.

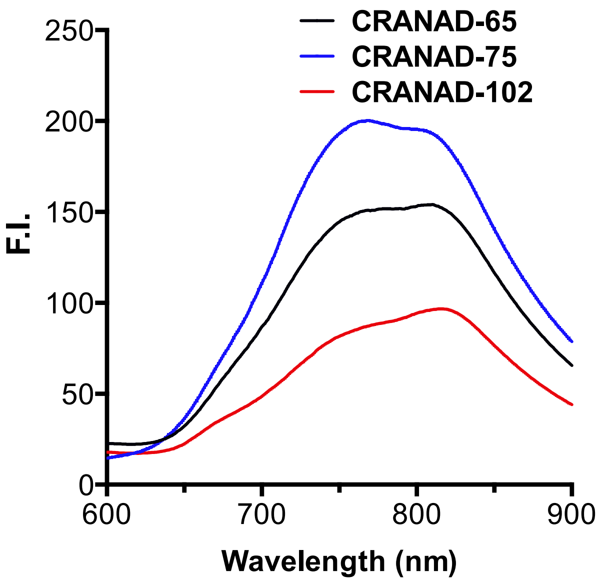

SI Fig. 2 Fluorescence emission spectra of CRANAD-65, -75 and -102 (Ex = 560 nm).

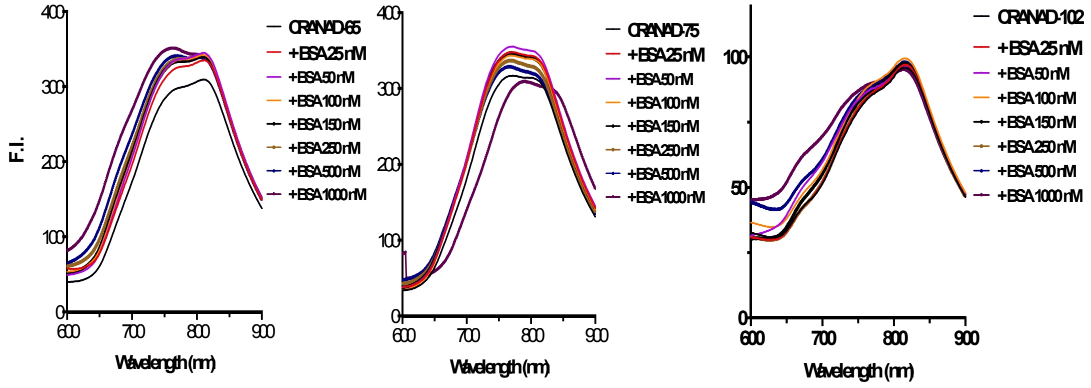

SI Fig. 3 Fluorescence emission spectra of CRANAD-65, -75, and -102 (250 nM) with different concentrations of Bovine serum albumin (BSA). No apparent changes of fluorescence properties with BSA could be observed.

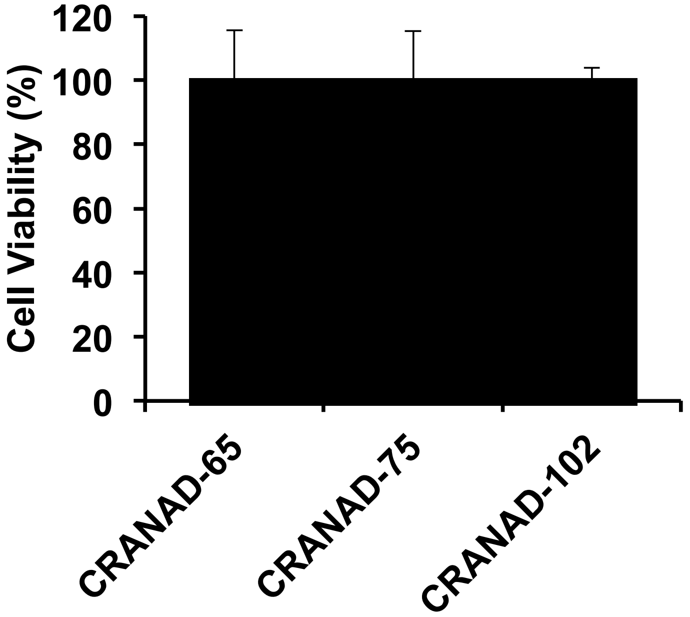

SI Fig. 4 Cytotoxicity studies of CRANAD-65, -75, and -102 with HEK 293 cells. No apparent decrease of viability of cells could be found with these probes (5.0 μM).

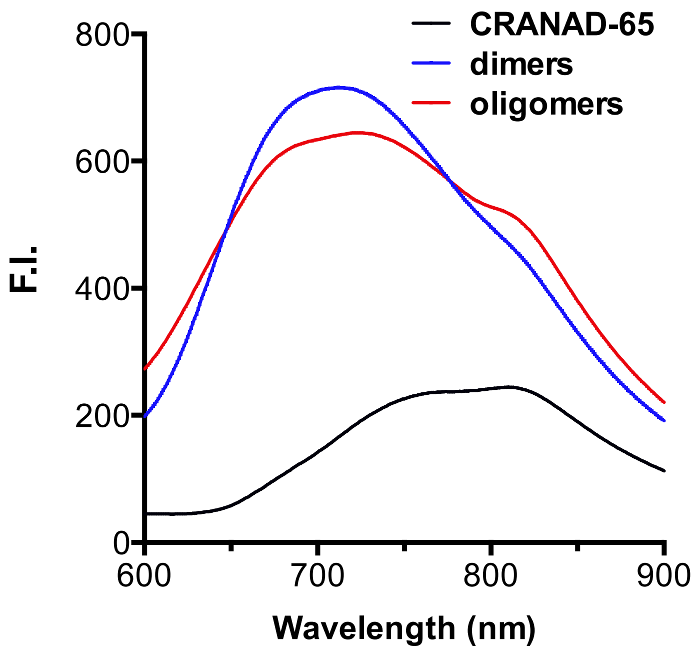

SI Fig. 5 Fluorescence emission spectra of CRANAD-65 (250 nM) with Aβ40 dimes and Aβ42 oligomers (750 nM).

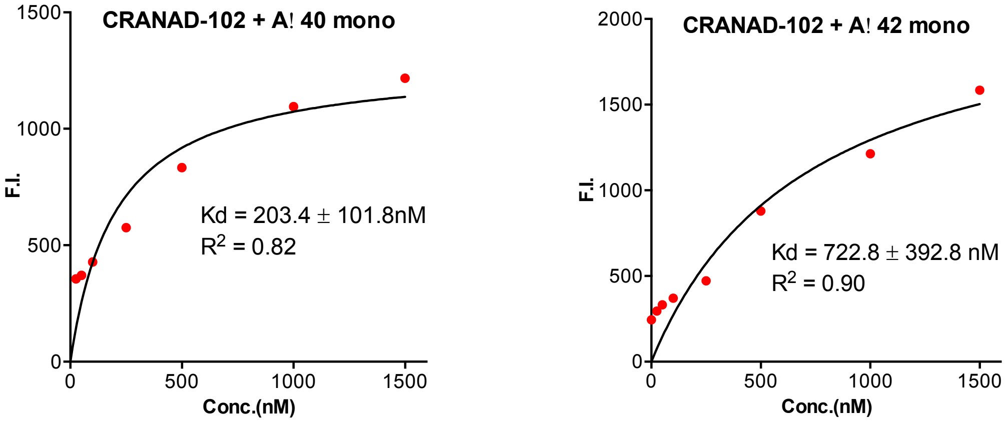

SI Fig. 6 Kd measurement of CRANAD-102 with Aβ40 monomers (left panel) and Aβ42 monomers (right panel)

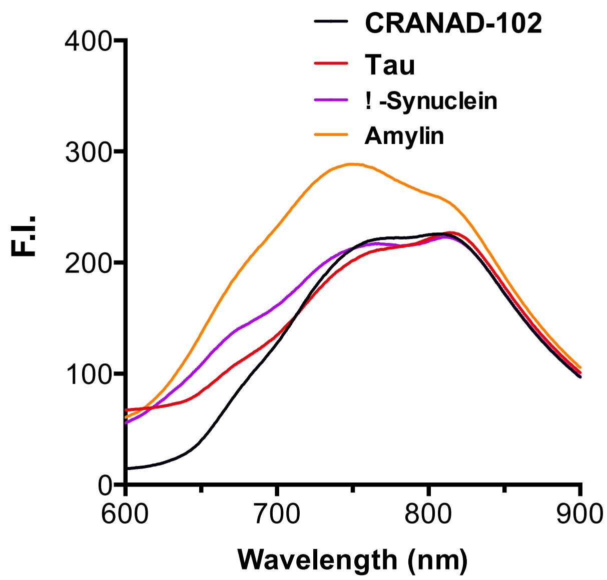

SI Fig. 7 Fluorescence emission spectra of CRANAD-102 (250 nM) with Tau 441, α-synuclein and amylin (750 nM).

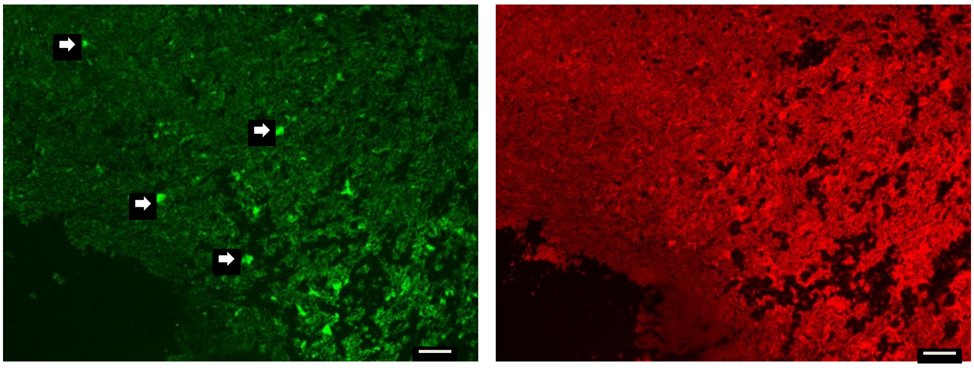

SI Fig. 8 Histological staining with Thioflavin S (left panel) and CRANAD-102 (right panel) for two consecutive brain slices of 14-month APP/PS1 mouse. Clear staining of plaques can be seen with Thioflavin S (white arrow), but no apparent plaque staining can be seen with CRANAD-102.

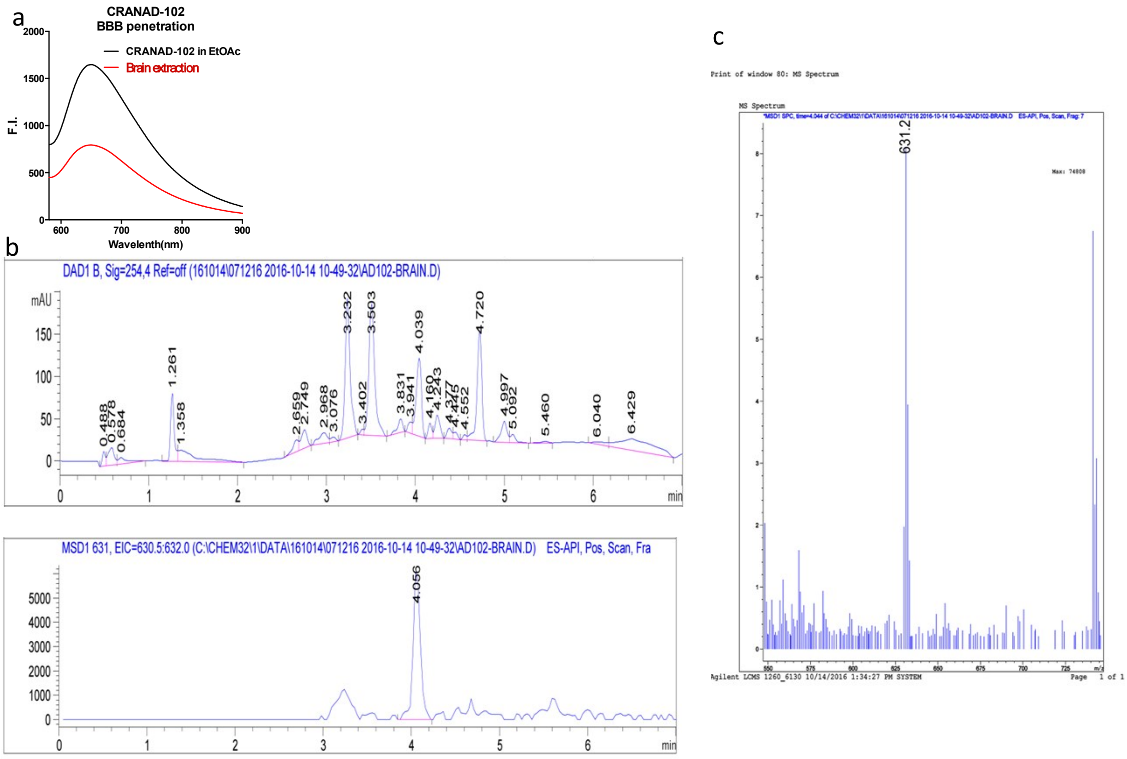

SI Fig. 9 BBB penetration stdudies of CRANAD-102. a) Fluorescence spectra of CRANAD-102 in Ethyl Acetate (EtOAc) (standard) and brain extraction with EtOAc after CRANAD-102 was iv injected. b) LC-MS of CRANAD-102 in brain extraction. Upper panel: LC spectrum of CRANAD-102, and bottom panel: XIC (extracted ion current) of CRANAD-102 with a molecular weight range of 630-632. c) MS spectrum of CRANAD-102 in brain extraction.

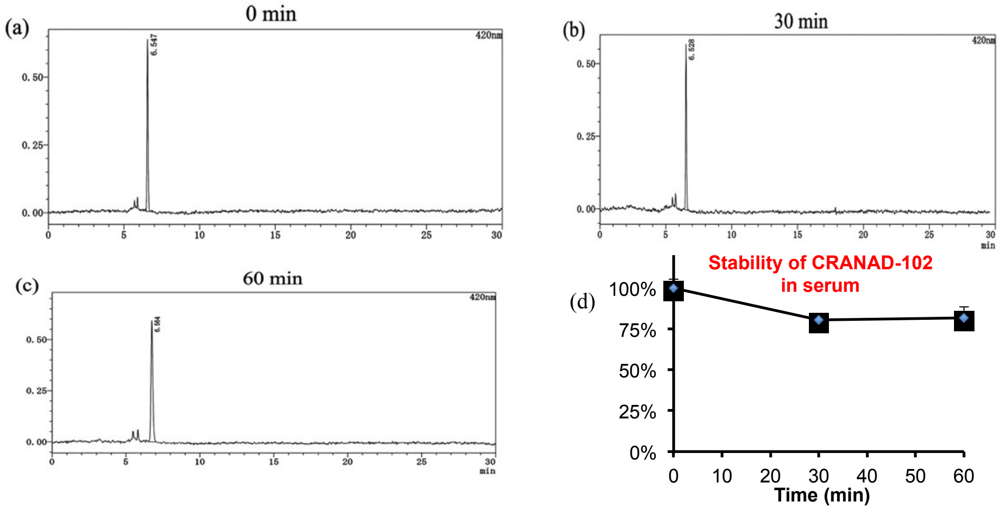

SI Fig 10. In vitro stability of CRANAD-102 in mouse serum at 37℃. (a - c): HPLC profiles of CRANAD-102 after incubation with mice serum for 0-, 30- and 60-min; (d) Quantification of HPLC peaks, and nearly 80% of CRANAD-102 was remained after incubating in mouse serum for 60-min.

3. ^{1}H NMR, ^{13}C NMR, HR-MS，HPLC

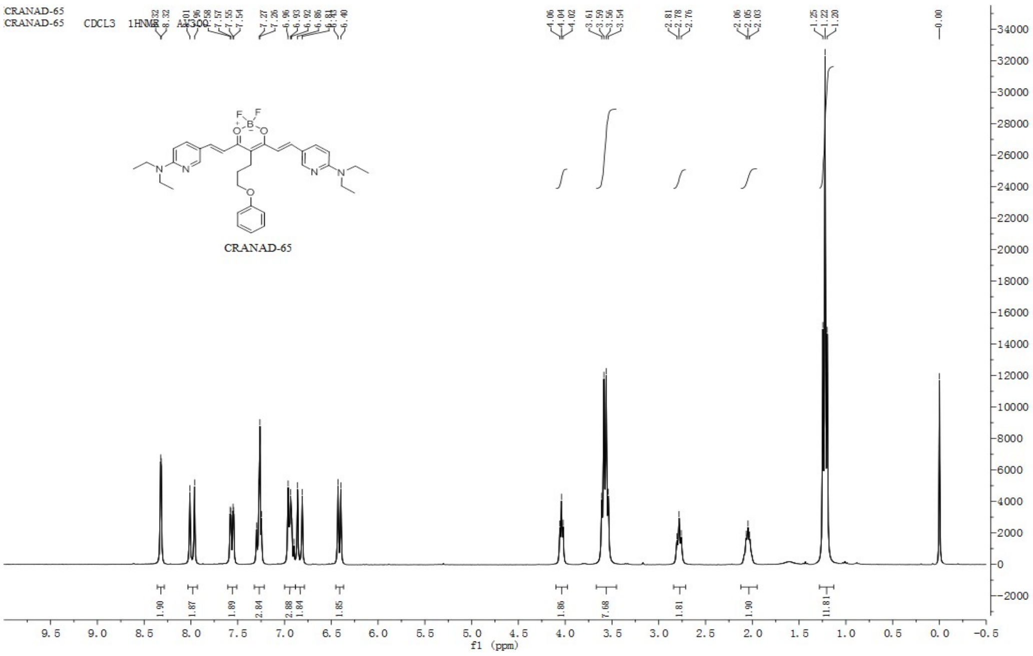

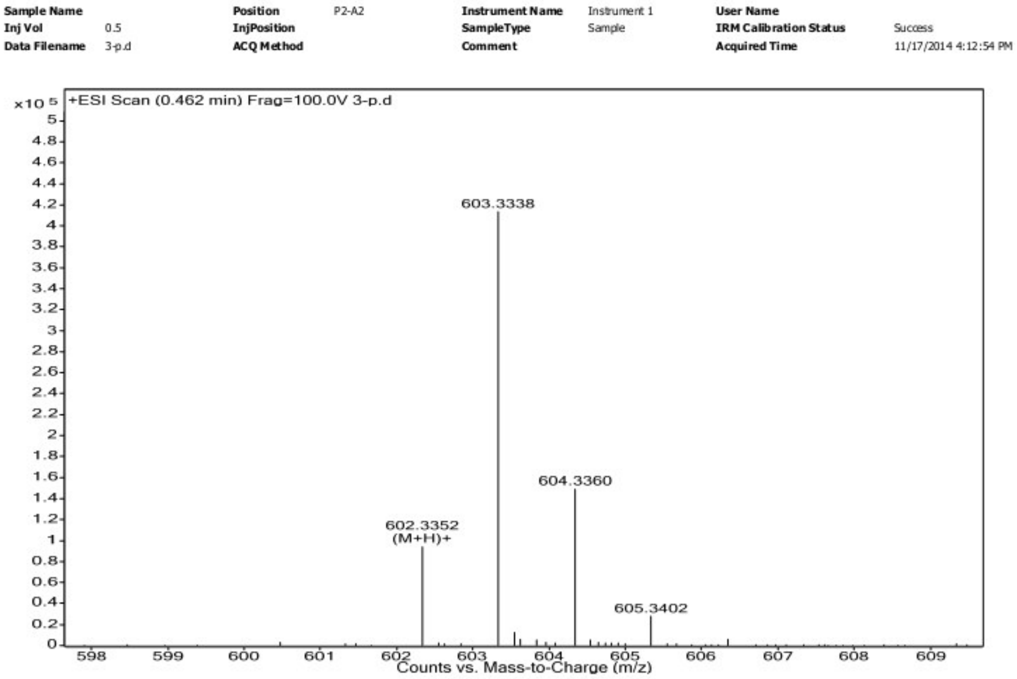

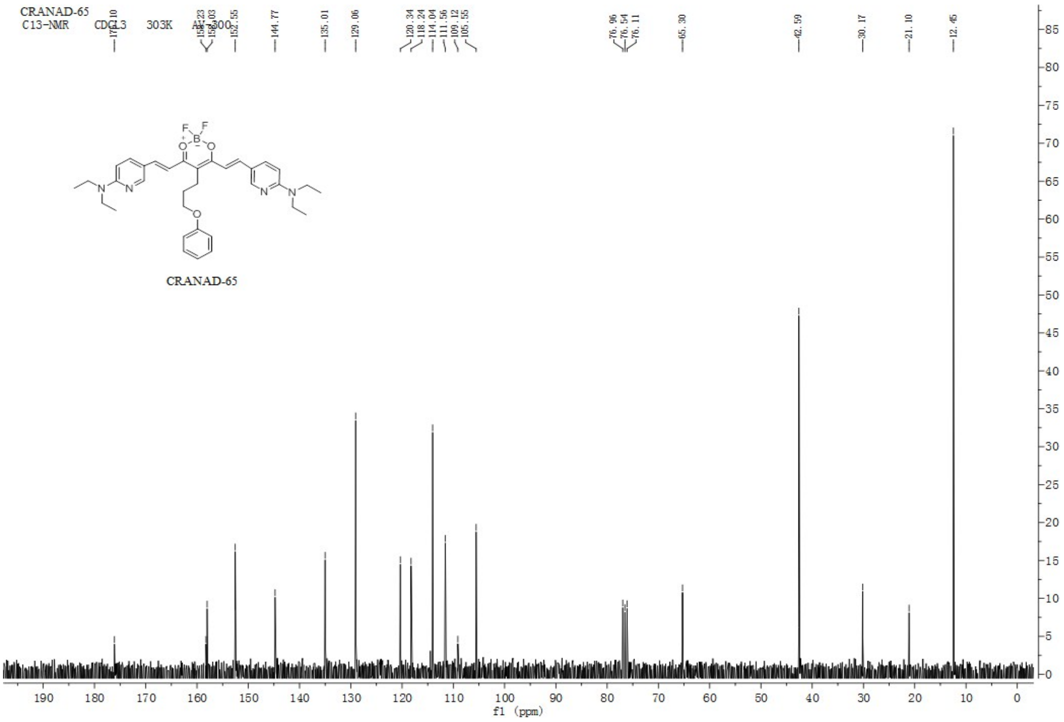

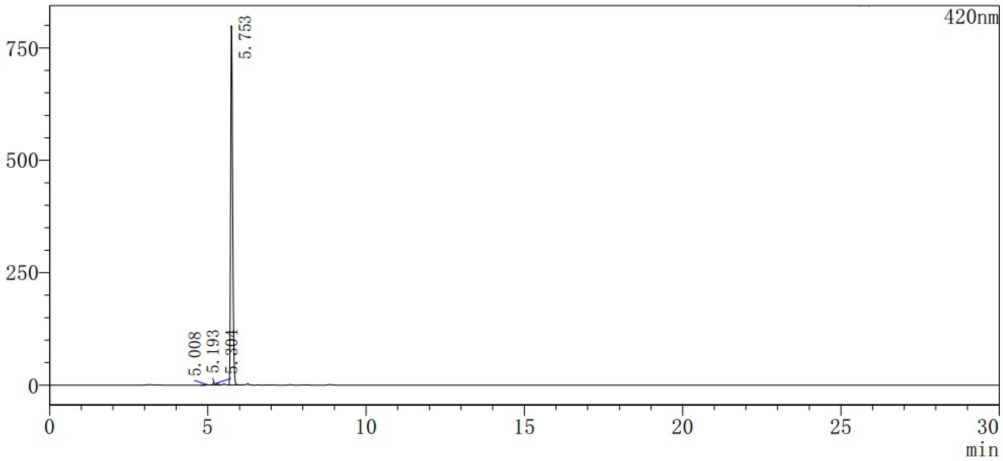

HPLC purity of compound CRANAD-65: 98.720%.

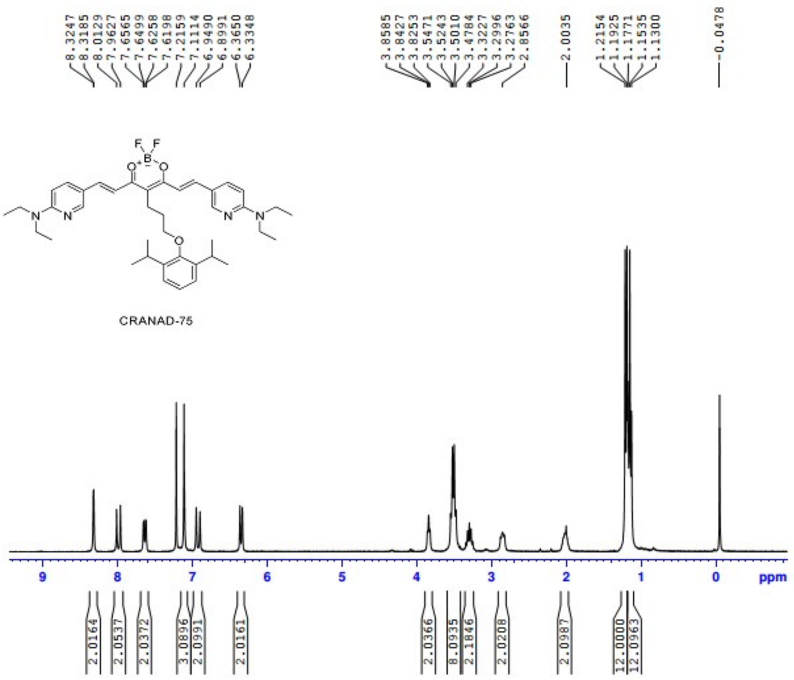

**Sample Name**		**Position**	P2-B3	**Instrument Name**	Instrument 1	**User Name**	
**Inj Vol**	2	**InjPosition**		**SampleType**	Sample	**IRM Calibration Status**	Success
**Data Filename**	18-5-p.d	**ACQ Method**		**Comment**		**Acquired Time**	4/20/2016 5:37:45 PM

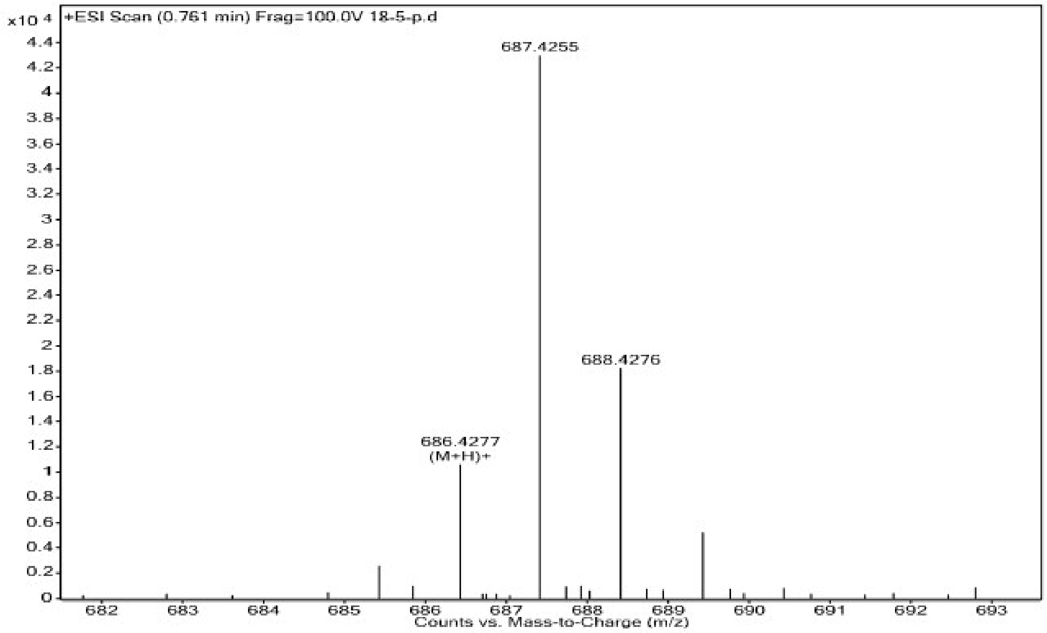

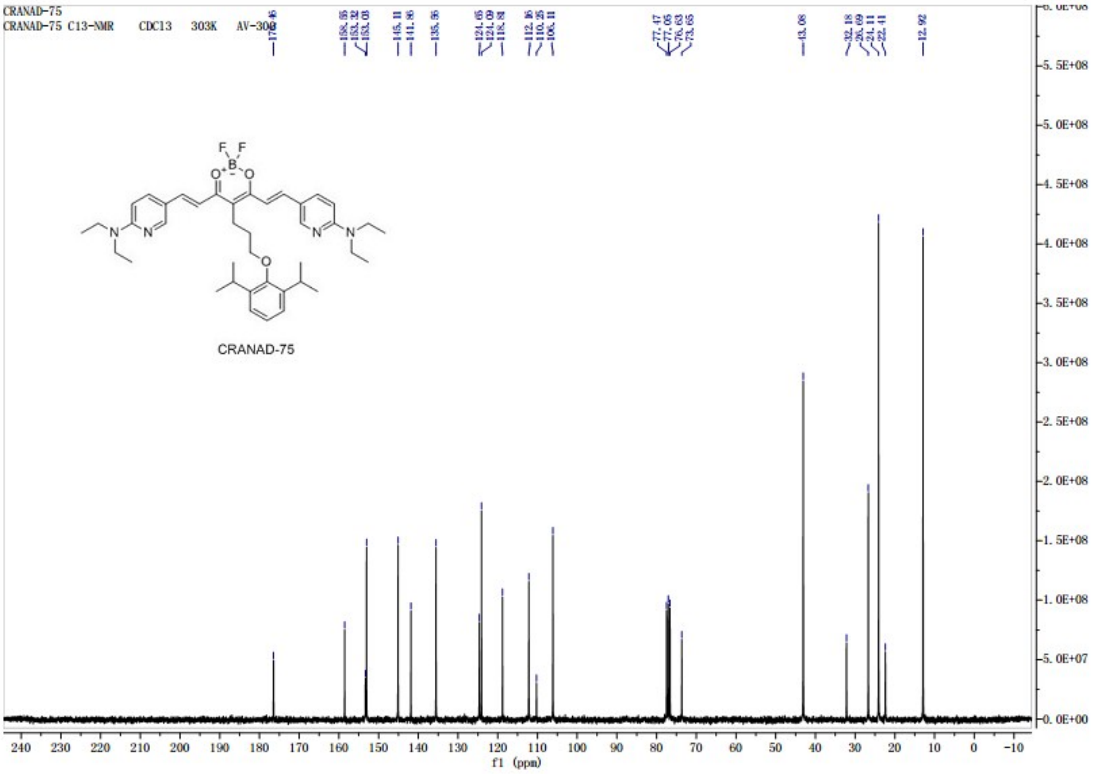

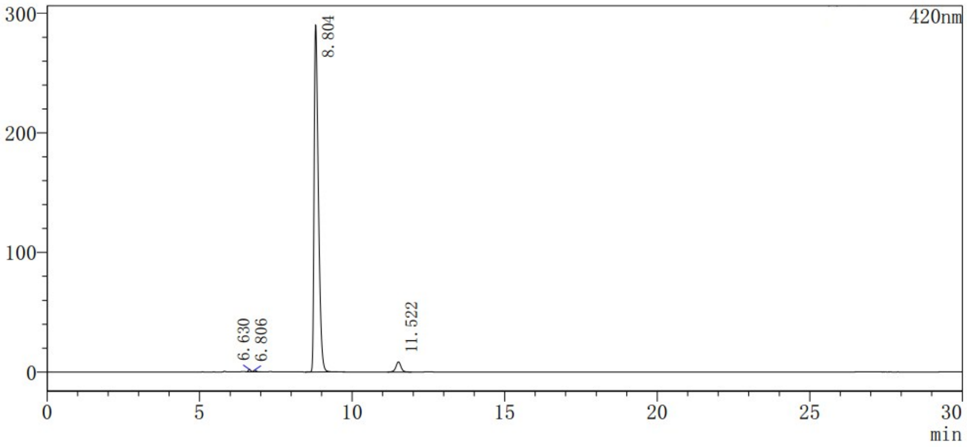

HPLC purity of compound CRANAD-75: 96.041%.

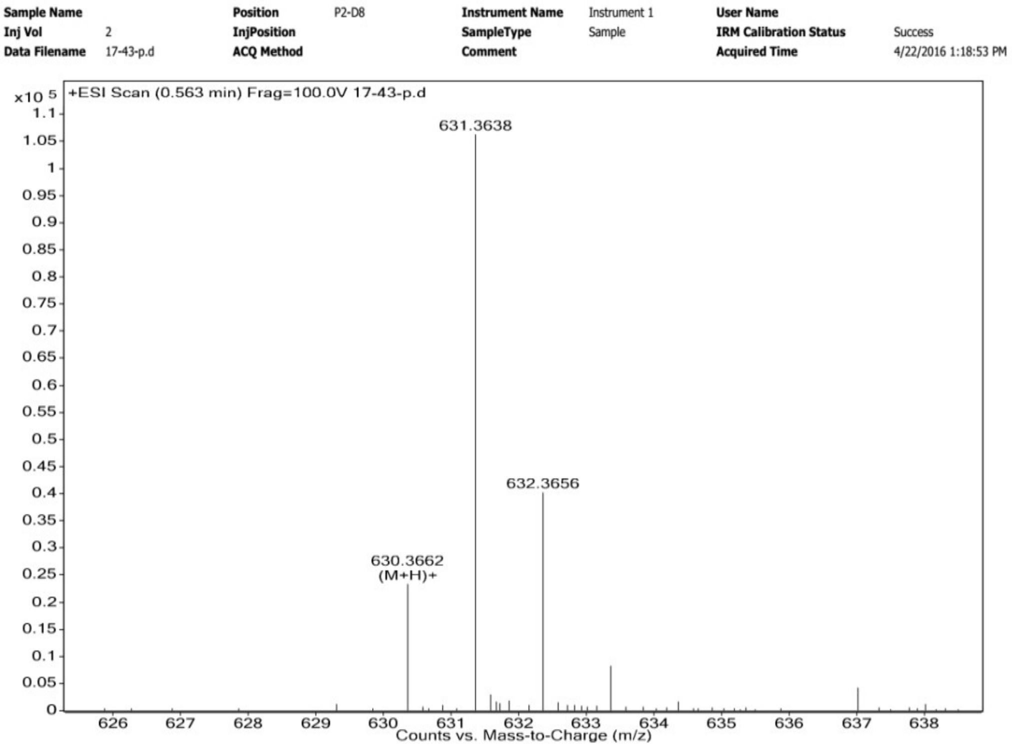

Sample Name
Position P2-D8
Instrument Name Instrument 1
User Name
Inj Vol 2
InjPosition
SampleType Sample
IRM Calibration Status Success
Data Filename 17-43-p.d
ACQ Method
Comment
Acquired Time 4/22/2016 1:18:53 PM
x10 5
+ESI Scan (0.563 min) Frag=100.0V 17-43-p.d
631.3638
632.3656
630.3662
(M+H)+
Counts vs. Mass-to-Charge (m/z)

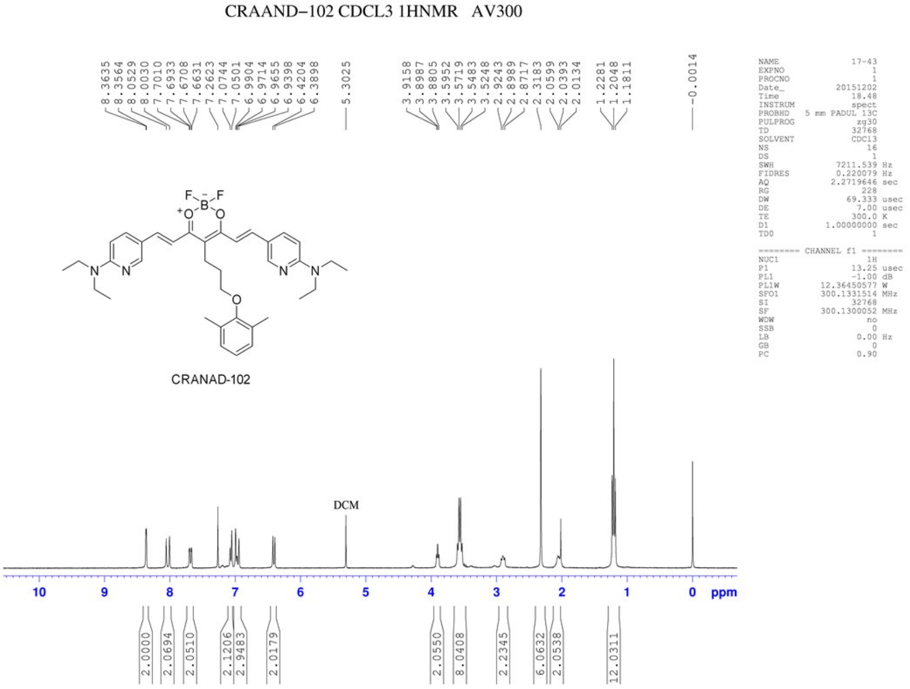

CRAAND−102 CDCL3 1HNMR AV300
CRANAD-102
DCM
ppm

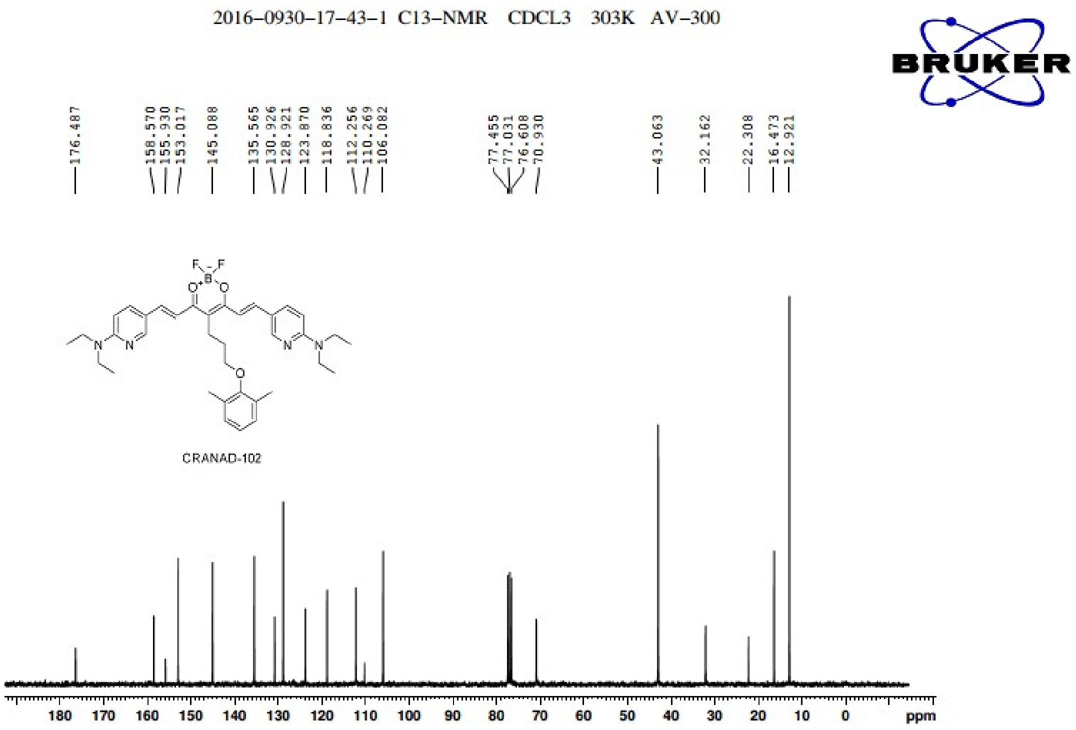

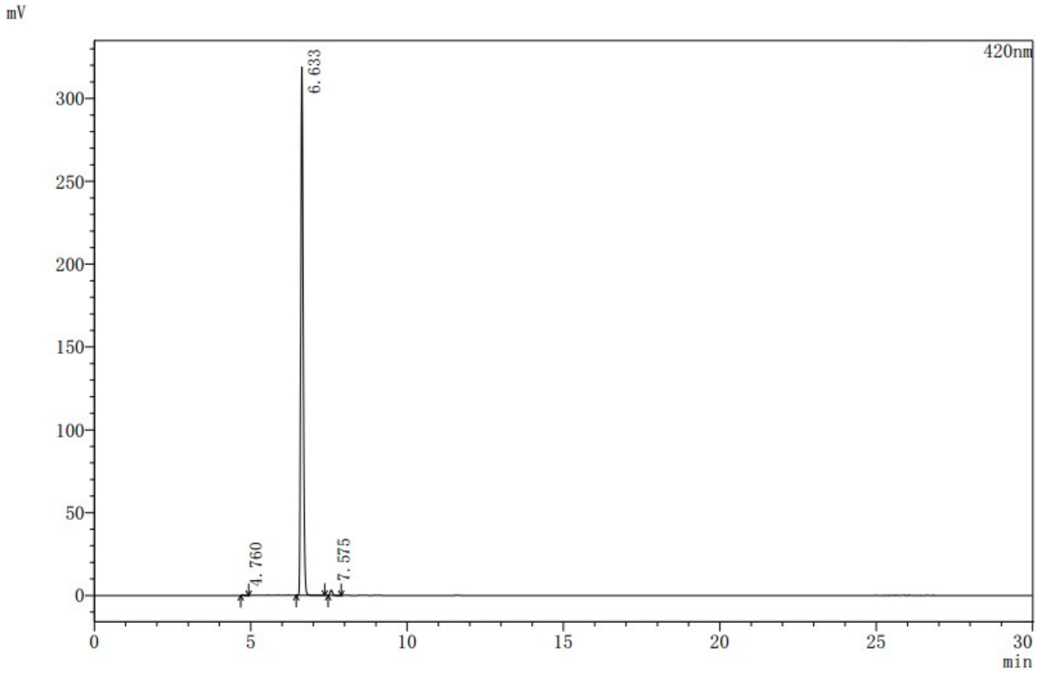

HPLC purity of compound CRANAD-102: 98.828%.
